# Effects of Annealing Temperature on the Crystal Structure, Morphology, and Optical Properties of Peroxo-Titanate Nanotubes Prepared by Peroxo-Titanium Complex Ion

**DOI:** 10.3390/nano10071331

**Published:** 2020-07-08

**Authors:** Hyunsu Park, Tomoyo Goto, Sunghun Cho, Soo Wohn Lee, Masato Kakihana, Tohru Sekino

**Affiliations:** 1 Department of Advanced Hard Materials, The Institute of Scientific and Industrial Research (ISIR), Osaka University, Osaka 567-0047, Japan; hspark23@sanken.osaka-u.ac.jp (H.P.); goto@sanken.osaka-u.ac.jp (T.G.); shcho@sanken.osaka-u.ac.jp (S.C.); kakihana@sanken.osaka-u.ac.jp (M.K.); 2 Department of Environmental and Bio-chemical Engineering, Sun Moon University, Asan 31460, Korea; swlee@sunmoon.ac.kr; 3 Institute of Multidisciplinary Research for Advanced Materials, Tohoku University, Sendai 980-8577, Japan

**Keywords:** titanate nanotubes, titanate nanosheets, titanate nanoplates, peroxo-titanium complex ion, annealing

## Abstract

This study addresses the effects of annealing temperatures (up to 500 °C) on the crystal structure, morphology, and optical properties of peroxo groups (–O–O–) containing titanate nanotubes (PTNTs). PTNTs, which possess a unique tubular morphology of layered-compound-like hydrogen titanate structure (approximately 10 nm in diameter), were synthesized using peroxo-titanium (Ti–O–O) complex ions as a precursor under very mild conditions—temperature of 100 °C and alkali concentration of 1.5 M—in the precursor solution. The nanotubular structure was dismantled by annealing and a nanoplate-like structure within the range of 20–50 nm in width and 100–300 nm in length was formed at 500 °C via a nanosheet structure by decreasing the specific surface area. Hydrogen titanate-based structures of the as-synthesized PTNTs transformed directly into anatase-type TiO_2_ at a temperature above 360 °C due to dehydration and phase transition. The final product, anatase-based titania nanoplate, was partially hydrogen titanate crystal in nature, in which hydroxyl (–OH) bonds exist in their interlayers. Therefore, the use of Ti–O–O complex ions contributes to the improved thermal stability of hydrogen titanate nanotubes. These results show a simple and environmentally friendly method that is useful for the synthesis of functional nanomaterials for applications in various fields.

## 1. Introduction

One-dimensional nanostructures, such as nanowires [1], nanobelts [2], nanorods [3], and nanotubes [4] which have received great attention due to their intriguing nanostructure and excellent properties [5]. Titania and titanate materials are nanostructures and they have excellent properties. One-dimensional nanostructured titania and titanate materials have attracted considerable attention and have been widely investigated in various fields such as environmental purification, photocatalysis, self-cleaning coatings, gas-sensor materials, electrode materials for dye-sensitized solar cells, as well as electron-transfer-layer (ETL) for perovskite photovoltaic cells [6,7,8,9,10]. This is due to their high redox potential, chemical stability, inexpensiveness, and non-toxicity [11]. The wide variety of their functional properties can be improved by the control of the nanostructure of titania and titanate. Nanotube structures [12], in particular, have received a lot of attention due to their large specific surface areas as well as internal and external surfaces, and their high porosity structures [13], which increase the number of potential active sites for a given reaction [14] that improves the catalytic properties and electricity conversion effects [15].

There are many routes for synthesizing titania/titanate nanotubes (TNTs), which include anodizing [16], sol-gel template synthesis [17], hydrothermal [4], and solution chemical synthesis methods [18] that are based on alkali treatment. Among these methods, the chemical synthesis route is relatively simple and facile. Although TNTs with an average diameter of 10 nm and a high specific surface area could be synthesized using this method, this process requires a temperature of 115 °C and duration above 24 h, as well as a high alkaline concentration above 10 M NaOH of the synthesis solution. However, the NaOH is classified as a toxic chemical by the Toxic Use Reduction Act. When used in large quantities, it poses significant chemical hazards to the environment or our health. Many groups have tried to modify this process and analyze the structure of TNTs, and they have reported that TNTs are composed of a layered titanate structure. They proposed that synthetic mechanism should be the dissolution/recrystallization process [19]. In many cases, titanate compounds such as porous crystalline titanate [20] or titanium alkoxides were used as starting materials for the synthesis of nanostructured titanates.

As mentioned earlier, the use of these raw materials requires severe synthesis conditions and high energy, which is due to the use of a large number of alkaline species for dissolving raw materials containing Ti. From the perspective of green chemistry, the use of titanium chelate complexes have also been reported [21,22,23]. This study introduces the novel bottom-up synthesis process for tubular structured titanate at a relatively low alkaline concentration of 1.5 M, using peroxo-titanium (Ti–O–O) complex ion as a precursor (named as PTNTs, peroxo-titanate nanotubes). In this way, this PTNTs can be also synthesized at a low temperature of approximately 100 °C using a novel bottom-up synthesis method, which is an environmentally-friendly process PTNTs possess a special peroxo-titanium bond (Ti–O–O) in the crystals as well as on the surface, which may enhance light absorption by reducing the optical band gap energy [24,25]. However, to apply this material to various engineering applications, it is very important to evaluate its thermal properties. For example, when applied as electrodes to dye-sensitized solar cells or chemical sensor devices, thermal treatment is required to make strong and stable oxide films/coatings, remove the binder, and for adhesion with substrates. Moreover, many reports demonstrated the TiO_x_ conducting layer annealed at high temperature so the electrons can be transferred through the valence band of the TiO_x_ [26]. However, the heating of nanostructured materials often affects the morphological change and the crystal structure due to sintering and chemical reactions [27,28]. In gas sensor applications as oxide semiconductors, the operating temperature for detecting the target gas is usually high and related to a variety of properties of the material such as the crystal structure, the particle size, and the energy band structure. Therefore, the evaluation of the effect of heating is essential for effectively setting the operating temperature and effective detection [29]. In the case of nanostructured titania/titanate synthesized at low temperature as described above, detailed understanding and clarifications on the relationship between the crystallographic properties induced by varying the operating temperature and the effects on functionality need to be considered.

Various studies have reported the structural changes of TNTs effected by thermal treatment [30,31,32,33,34]. Zhang et al. [34] reported the formation of the anatase phase by heating at a temperature above 500 °C and the change in nanotubular morphology to a spherical shape. We reported that the synthesis of TNTs following Kasuga’s method preserved the nanotubular morphology and maintained its high surface area, but it was transformed to an anatase-type TiO_2_ at 450 °C, while a sudden decrease in the surface area and morphological change to a spherical shape was confirmed by further heating above 450 °C [33]. PTNTs contain peroxo groups (–O–O–) that modify the chemical bonding nature of Ti–O and the resultant crystalline structures. Therefore, PTNTs are expected to show different thermal stability, as well as crystallographic and morphological changes compared to the commonly known titania/titanate nanotubes because of its unique structure with the peroxo group. In addition, understanding the thermal properties of TNTs is an important factor for designing TNTs’ functions and applications. Etacheri et al. [11] reported the high-temperature stability of oxygen-rich titania synthesized by a peroxo-titania complex. However, there are few studies on the effects of heat treatment on the morphological and crystallographic characteristics, as well as physical/chemical properties of peroxo-titanium containing nanostructured titanate, especially nanotubular titanate (thus PTNTs). It is necessary to develop an effective method for the synthesis of titanate nanostructures with good visible light responsibility even after the heating at high temperatures.

The purpose of this study is thus to clarify the relationships between the heat treatment of the crystalline structures and the morphology of the titanate nanotubes containing PTNTs that was prepared from peroxo-titanium complex ions at a temperature as low as 100 °C. In situ high-temperature X-ray diffraction was used to observe the crystallographic changes induced by the thermal treatment of PTNTs. The effect of annealing temperature on the morphological and optical characteristics of PTNTs is discussed using physicochemical investigations.

## 2. Materials and Methods

### 2.1. Synthesis of Materials

As a precursor, peroxo-titanium complex ion [35] was synthesized by the following method: Initial 0.63 g of TiH_2_ (> 99%, Kojundo Chemical Laboratory Co., Ltd., Saitama, Japan) was dissolved in 62.46 mL of mixed solution with a pH of 10 containing H_2_O_2_ (30%, FUJIFILM Wako Pure Chemical Corporation, Osaka, Japan) and NaOH (97%, FUJIFILM Wako Pure Chemical Corporation, Osaka, Japan) aqueous solution for 2 h at 10 °C. The pH of the mixed solution was controlled by gradually adding 10 M of concentrated NaOH solution to the hydrogen peroxide solution. Consequently, the Na concentration in the precursor solution was maintained at 1.5 mol/L (1.5 M) and the last step is to add alkaline (Na) to synthesize nanotubular titanate. It should be noted that the necessary amount of Na required for the synthesis of TNTs in this study was much less compared to the commonly known chemical synthesis process, which is typically 10 M [18]. To synthesize peroxo-titanate nanotubes, the prepared peroxo-titanium complex ion solution was put in a polytetrafluoroethylene bottle equipped with a reflux condenser and it was placed in an oil-bath. It was heated at 100 °C for 12 h with and it was stirred at string speed of 200 rpm. Afterward, the precipitates were treated using a 5 M HCl (30%, FUJIFILM Wako Pure Chemical Corporation, Osaka, Japan) solution until the pH became 5, and it was washed using distilled water and a vacuum pump (MDA-020C, ULVAC, Inc., Kanagawa, Japan) until the ion conductivity of the filtered solution became less than 5 µS/cm. The product was dried using a freeze dryer (EYELA FDU-2200, TOKYO RIKAKIKAI CO, LTD., Tokyo, Japan) and was labeled as PTNTs. Afterward, to investigate the effect of heating on PTNTs, the samples were thermally treated at 200, 300, 400, and 500 °C using a furnace. All samples were heated under ambient conditions at 10 °C/min and kept at a given temperature for 1 h.

### 2.2. Characterization

The thermal properties of the PTNTs were studied using thermogravimetric differential thermal analysis (TG-DTA, TG8120, RIGAKU, Tokyo, Japan). Approximately 10 mg of the sample was weighed on a Pt pan and the α-alumina powder was used as the reference material. Scans between 25 and 600 °C were carried out at a heating rate of 10 °C/min under ambient conditions. The temperature of the instrument was calibrated by using purity standard metallic materials (In, Pb and Au). The morphology and particle size were observed using field-emission scanning electron microscopy (FE-SEM, SU-9000, Hitachi High-Technologies Corporation, Tokyo, Japan) with 0.4 nm resolution at 30 kV acceleration voltage. The specific surface area was calculated using the Brunauer–Emmett–Teller (BET) method based on the adsorption isotherm at a P/P_0_ range of 0.1–0.3 using an N_2_ adsorption-desorption instrument (NOVA 4200e, Quantachrome Instruments, Boynton Beach, FL, USA). The crystalline phase was identified using X-ray diffraction (XRD, D8 ADVANCE, Bruker AXS Co. Ltd., Karlsruhe, Germany), and the diffraction patterns of each sample were collected using a vertical goniometer equipped with a high-temperature reaction chamber (XRK-900, Anton Paar, Graz, Austria), operating in the Bragg configuration using Cu Kα radiation (λ = 1.54056 Å) from 5 to 85° at a scanning rate of 0.02°. In-situ XRD studies were recorded during the annealing process, heating occurred from 25 to 560 °C at a rate of 10 °C/min. From the results of the XRD analysis, the lattice parameters of the samples were evaluated using Equation (1).

(1) $$\frac{1}{d^{2}}=\frac{h^{2}}{a^{2}}+\frac{k^{2}}{b^{2}}+\frac{l^{2}}{c^{2}}$$

where, *d* is the interplanar spacing and *h, k, l* are Miller indices. *a, b*, and *c* are lattice constant of crystal phase. The anatase crystallite size of samples was evaluated using Equation (2).

(2) $$D=\frac{0.94\lambda }{\beta cos\theta }$$

where D is the crystalline size of materials, *λ* is the wavelength of X-ray, *β* is the broadening of the diffraction line measured at half of its maximum intensity in radians, and *θ* is the angle of diffraction. The shape factor of 0.94 was used by assuming a sphere shape in this study. Transmission electron microscopy (TEM, JEOL-2100, JEOL, Tokyo, Japan) was performed at an acceleration voltage of 200 kV to further characterize individual nanostructures of titanate or titania. The lattice spacing, fast Fourier transform (FFT), and the phase interpretation were investigated using the Gatan Digital Micrograph software [36] (Gatan Inc., Pleasanton, CA, USA). Fourier transform infrared spectrometer (FT-IR, FT/IR4100, JASCO, Tokyo, Japan) spectra were obtained within the range of 4000–500 cm^−1^ region at a resolution of 4 cm^−1^. The samples were measured using the KBr pellet method in the transmission mode. Raman spectra were acquired with a Raman micro-spectrometer (HR 800, HORIBA, Kyoto, Japan) using an Ar ion laser (514.5 nm). The spectra were collected in the range of 1000–100 cm^−1^ with a resolution of 1 cm^−1^. The reflectivity of the prepared powder was evaluated using the solid sample measurement mode in the ultraviolet-visible (UV-Vis) machine. Diffuse reflectance spectroscopy (DRS) (V-650, JASCO Co., Tokyo, Japan) and optical band gap energy were measured using the Tauc-plot method using Kubelka–Munk transformation. The powders were uniformly pressed in a powder holder and placed in the sample holder on an integrated sphere for the reflectance measurements. X-ray absorption near edge structure (XANES) and extended X-ray absorption fine structure (EXAFS) spectra of the Ti K-edge were measured in an ionization chamber in transmission mode on the Kyushu University Beamline (BL06) of the Kyushu Synchrotron Light Research Centre (SAGA-LS; Tosu, Japan). Pellet samples for Ti K-edge XANES and EXAFS measurements were prepared as mixtures with boron nitride. Anatase (TiO_2_, FUJIFILM Wako Pure Chemical Corporation, Osaka, Japan) was used as a reference sample. Ti K-edge spectra were collected over a photon energy range of 4635.2–5938.6 eV, and the spectra were analyzed using ATHENA software [37] (Version 0.9.26, Ravel and Newville 2005,).

## 3. Results and Discussion

### 3.1. Thermal Properties of Materials

The powdered products were successfully synthesized following the chemical processing where peroxo-titanium complex ion solution is used as a precursor for the synthesis of nanostructured titania. The powder was yellow, and the optical bandgap energy was greatly reduced to 2.44 eV when compared with the white color of pure TNTs with an optical band gap of 3.34 eV. The yellow-colored TiO_2_-based compounds have been synthesized by anion doping such as N [38] and cation doping like Nb [39]. However, the peroxo-titanium complex ion precursor contains only H, O, Na and Ti in solution, and thus the bottom-up process in this study does not include any doping elements like N and Nb that introduce formation of impurity levels among the band gap in TNTs. Therefore, this yellow color is considered to be caused by the reduced band gap of titanate without any doping. In addition, this implies that the present processing method that makes use of precursor complex ions and following the chemical route might be a facile but promising method for the production of functionalized oxides with low-dimensional nanostructures.

**Figure 1:**
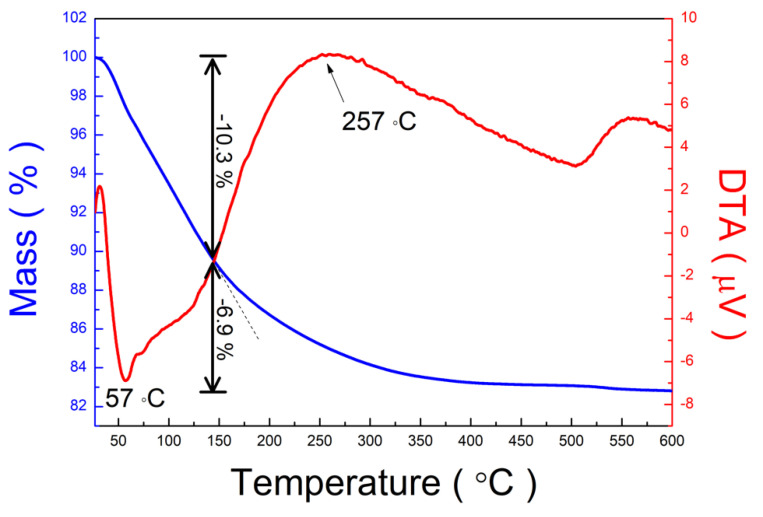
Thermogravimetric (TG) and differential thermal analysis (DTA) curves of as-synthesized peroxo-titanate nanotubes (PTNTs) heated at 10 °C/min in an air atmosphere.

The thermal properties of the prepared PTNTs in the temperature range of 25–600 °C were characterized by TG and DTA, as shown in Figure 1. The total weight loss was approximately 17.2%, and the significant weight loss was observed in the temperature range of 25–143 °C. Within this range, 10.3% of weight loss was estimated to be due to the desorption of molecular H_2_O such as dissociated H_2_O, physisorbed H_2_O, and chemisorbed H_2_O [32]. This weight loss can also be attributed to the structural water loss of the titanium oxide structure including the Ti–OH bond [40,41]. An endothermic peak was observed at approximately 57 °C in the DTA curve due to water evaporation of physically adsorbed water on the PNTs. Subsequently, a continuous weight loss of 6.9% was obtained at 600 °C, which may also be assigned to structural water loss, resulting in the transformation of the crystal structure [32]. An exothermic peak is observed in the DTA graph due to the transformation of a crystal structure at approximately 257 °C.

### 3.2. Morphology of Materials

**Figure 2:**
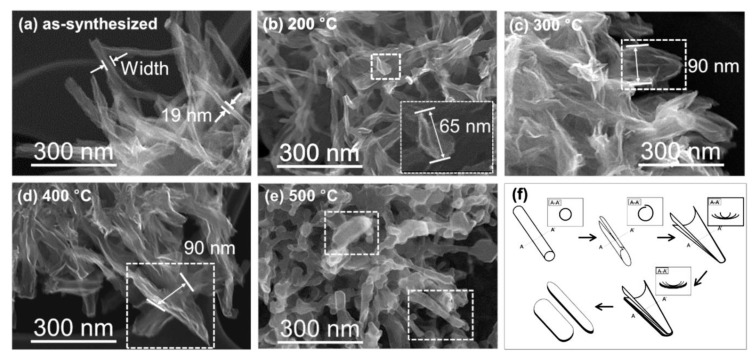
Scanning electron microscopy (SEM) images of (**a**) as-synthesized PTNTs and (**b**–**e**) annealed PTNTs at 200, 300, 400 and 500 °C for 1 h, respectively. (**f**) Schematic description of the annealing temperature effect on the morphologies of PTNTs.

The SEM images of prepared samples are shown in Figure 2. The as-synthesized samples had tubular structures, as shown in Figure 2a. The average diameter of tubular structures was found to be approximately 10–20 nm, and after heating at 200 °C, distorted tubular structures were often observed (Figure 2b). At the heating temperature of 300 °C, sheet-like structures were frequently seen, as shown in Figure 2c. The sheet-like structures seem to stack on each other, and these stacked sheet-like structures were still observed in the sample that was heated up to 400 °C (Figure 2d). Further annealing at 500 °C resulted in the formation of a plate or rod structures, as shown in Figure 2e. This was observed not only in the specific part among the agglomerates in the samples but also in the whole part of each sample.

Figure 2f shows the schematic diagram of the morphology change caused by the increase in annealing temperatures, which mainly represents the typical structures at each temperature, as shown in dotted boxes in each SEM image (Figure 2a–e). The inset in Figure 2f represents the expected cross-section of each structure. The as-synthesized PTNTs had a tubular structure. This tubular structure seems to be “dismantled” above 200 °C of annealing, and at approximately 200 °C, the end part of the tube seems to re-open by forming a cleavage that seems to extend further. At 300 and 400 °C, “stacked bamboo leaf-like nanosheets” were frequently observed which might be formed by the re-opening of PTNTs. Although the structure, composition, and formation mechanism of titania nanotubes synthesized by chemical treatment methods are still under review [42], a nanotube structure originated from the characteristics of the layered hydrogen titanate crystal [13]. Thus, the TNTs are considered to be formed by the scrolling of titanate nanosheets to make a multi-wall titanate nanotube. Considering these facts, the formation of stacked leaf-like nanosheets by the re-opening of nanotubes, as shown in Figure 2c,d as well as the illustration in Figure 2e, may be feasible. From these perspectives, the phenomenon of the deformed tubular structure may imply a return to a sheet structure as a result of an increase in the annealing temperature. The annealing temperature at 300 °C causes the tubular structures to change into stacked sheet-like structures, and the sheet structure was assumed to show unstable surface energy due to its large surface area. However, the layer structure was gradually distorted due to the removal of water molecules from PTNTs by the annealing process as shown by the TG-DTA results (Figure 1). Therefore, it would be built up together to minimize its surface energy at a higher temperature (approximately 400 °C). As a result, transformation into a plate-like or rod-like structure consisting of bamboo leaf-like nanosheets stacks with a long aspect ratio was formed at a higher temperature that was up to 500 °C as surrounded by square dotted frames in Figure 2e as well as illustrated in Figure 2f. The fundamental mechanism of how the heating or thermal activation would make such a structural variation in the PTNTs has not been clarified yet, and thus the further detailed investigations are required through various in situ analysis using transmission/scanning transmission electron microscopy (STEM/TEM), thermal analysis coupled with mass spectroscopy and Fourier transform infrared (FT-IR) spectroscopy etc. However, we inferred that the decomposition of peroxo functional groups (–O–O–) and/or release of H_2_O might contribute to the systematic structure degradation of layered titanate nanotubes because such structure degradation has not been reported yet for the pure titania/titanate nanotubes.

**Figure 3:**
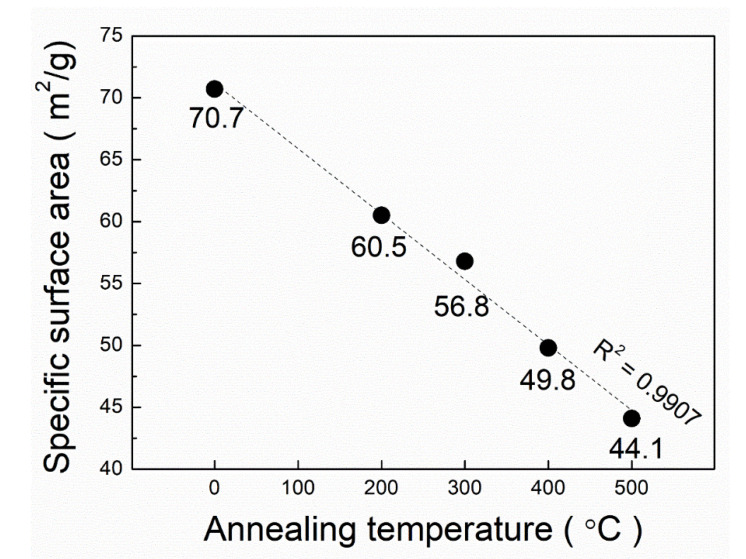
Variation of the specific surface areas of PTNTs annealed at 200, 300, 400, and 500 °C for 1 h, respectively.

Figure 3 shows the specific surface area (SSA) at various annealing temperatures. SSA decreased linearly (R-squared value = 0.9907) as the annealing temperature increased. In this study, the annealing temperature affected the morphology of the structure as observed in Figure 2. In this study, the surface area decreased, and it is expected that the dehydration of the surface of the structures caused agglomeration between each structural unit (i.e., individual nanotubes) and this reduces the specific surface area because the morphology and crystal structure were affected at higher temperatures [34], which was confirmed by the findings from the TG-DTA analysis shown in Figure 1.

### 3.3. Crystallographic Characteristics of Materials

**Figure 4:**
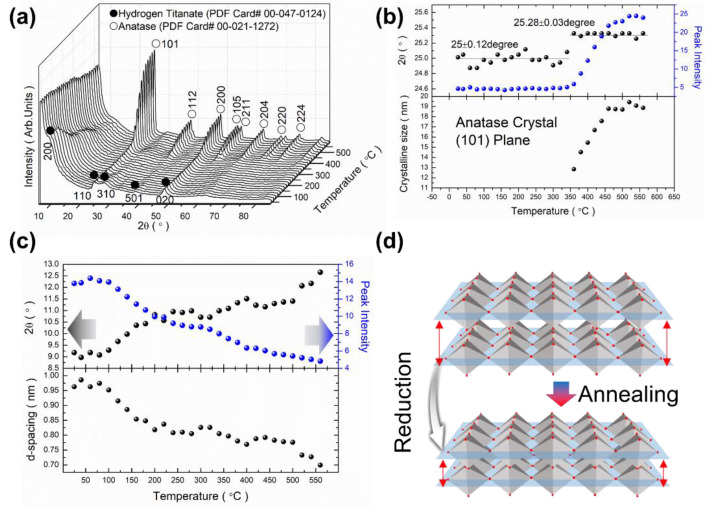
(**a**) In situ X-ray diffraction (XRD) patterns of the PTNTs during an annealing temperature. (**b**) Annealing temperature effect on the variations of 2θ and Peak intensity assigned to the 110 hydrogen titanate and 101 anatase reflections, and the crystal size of the anatase phase. (**c**) Annealing temperature effect on the variations of 2θ and peak intensity assigned to the 200 hydrogen titanate reflection and *d*-value derived from 2θ. (**d**) Illustration for variation of interlayer distance by annealing.

The crystalline phase variation of the PTNTs caused by heating was analyzed by in situ high-temperature X-ray diffraction, and the results are shown in Figure 4a. The as-synthesized PTNTs show a typical hydrogen titanate phase [43] (Powder Diffraction File (PDF) Card# 00-047-0124) with an orthorhombic structure. Titanate nanotube (H_2_Ti_2_O_5_·H_2_O) is considered to be formed by scrolling layers of titanate nanosheets and thus forming a layered-structure that corresponds to the 200 reflections of TNTs. The diffraction peaks related to the *c*-axis (e.g., 501, 002) were much weaker than the peaks at 200, 110, 310, 020 [44]. As the annealing temperature increases, the crystal phase is converted from hydrogen titanate to an anatase-type (PDF Card# 00-021-1272) with tetragonal structure. The lattice constants of reported H_2_Ti_2_O_5_·H_2_O [43] were: *a*_0_ = 18.03 Å, *b*_0_ = 3.784 Å, and *c*_0_ = 2.998 Å (PDF Card# 00-047-0124), and the lattice constants of the reported anatase were: *a*_0_ = 3.785 Å, *b*_0_ = 3.785 Å, and *c*_0_ = 9.514 Å (PDF Card# 00-021-1272). The lattice constants of anatase obtained after heating to 500 °C were: *a*_0_ = 3.764 Å, *b*_0_ = 3.764 Å, and *c*_0_ = 9.483 Å. The lattice constants were calculated using Equation (1) and the values of the as-synthesized PTNTs were *a*_0_ = 19.27 Å, *b*_0_ = 3.743 Å and *c*_0_ = 2.976 Å. Among the 3 parameters, the *b*_0_ and *c*_0_ values agreed with those of the base hydrogen titanate structure. However, the *a*_0_ value, which relates to the interlayer spacing of the titanate structure increased by 6.9%, which is more than that for the common hydrogen titanate. The large increase in the *a*-axis may be due to the enlargement of interlayer distance at the 200 plane. Sugita et al. [45] reported an increase in the interplanar spacing of titanate when the radius of the Li^+^ ions was greater than that of H^+^ ions located between the hydrogen titanate layers. Kong et al. [24] obtained similar results when a peroxo-titanium bond (Ti–O–O–H) was present between the layers of hydrogen titanate crystals.

The variations of each peak position (2θ), and the intensity assigned to 110 diffraction peaks of PTNTs (hydrogen titanate), the 101 diffraction peak of anatase phases, and the crystallite size with the annealing temperature of samples are displayed in Figure 4b. The 2θ angle of the diffraction peak assigned to 110 of hydrogen titanate crystal was maintained at 25.00° ± 0.12° till the heating temperature was up to 350 °C. Then, the peak position quickly shifted to a higher angle at the subsequent temperature range, and the 2θ value was 25.28° ± 0.12° which is the similar peak position of 101 in anatase TiO_2_ phase (calculated as 25.281° from the *d*-value). At the same time, as the temperature increased, the intensity of peak maintained a low value till the temperature was 350 °C and it linearly increased to 460 °C from the subsequent annealing temperature where it maintained its maximum value. The crystallite size of the anatase phase was calculated at 350 °C to be approximately 13 nm, which then increased to 19 nm as the annealing temperature increased up to 460 °C were maintained its highest size.

The change in the peak was also observed at the diffraction peak according to the 200 of hydrogen titanate. Figure 4c shows the variations in the peak position (2θ, intensity, and *d*-value of the 200 plane depending on the annealing temperature. No noticeable change was observed at the diffraction peak of 200 until a temperature change of 100 °C was attained. As the annealing temperature increases, the peak position shifted to a higher 2θ angle with a decrease in the peak intensity. As earlier mentioned, this 200 peak was assigned to the interlayer of hydrogen titanate, for which the interlayer distance of PTNTs could be said to decrease with the heating, as shown in Figure 4d. From the TG-DTA analysis in Figure 1, the heating at low-temperature up to approximately 120 °C, in which the major phenomenon was the removal of physically-adsorbed water from the surface, did not affect the structural characteristics of PTNTs. Furthermore, subsequent water loss could be attributed to the interlayer water loss as the peak shifted to a high angle, which indicates a reduction in the interlayer distance, as observed in XRD results. Our results show that the interlayer distance of approximately 0.9 nm in the as-synthesized PTNTs was maintained at 120 °C, followed by a rapid decrease in the distance within a temperature range of up to 200 °C. Then, as the annealing temperature further increases, the interplanar distance decreases linearly which results in a reduction of d = 0.7 nm at 560 °C.

### 3.4. Transmission Electron Microscopy

**Figure 5:**
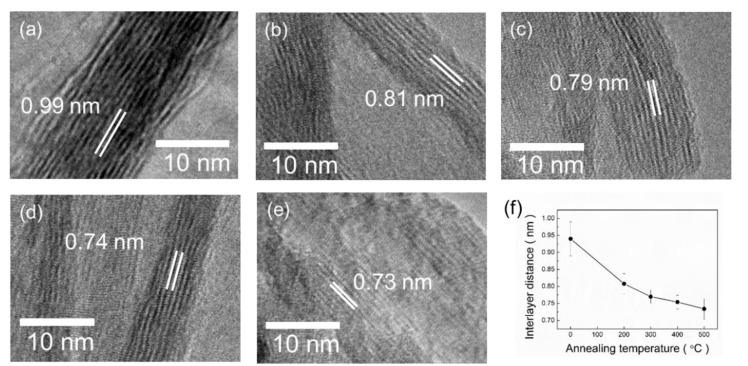
Transmission electron microscopy (TEM) images of (**a**) as-synthesized PTNTs and (**b**–**e**) annealed PTNTs at 200, 300, 400, and 500 °C for 1 h, respectively. (**f**) Variation in the interlayer distance for annealing temperatures taken from 30 zones, respectively.

To confirm the interlayer distance and distribution of the crystallites of PTNTs, high-resolution (HR) TEM observation was performed for the samples obtained at different annealing temperatures, as shown in Figure 5. All the samples show a periodic lattice image that corresponds to the 200 plane of layered hydrogen titanate, which was also observed from XRD analysis results (Figure 4). The interlayer distance was calculated to be 0.99 nm for the as-synthesized PTNTs (Figure 5a), and it clearly decreased with an increase in annealing temperature to 0.73 nm for PTNTs annealed at 500 °C (Figure 5e). The interlayer spacing obtained is summarized in Figure 5f, and the observed results were consistent with that calculated from the XRD analysis (d-spacing in Figure 4c). Their results were compared and replotted as Figure S2, which clearly exhibited similar tendency.

**Figure 6:**
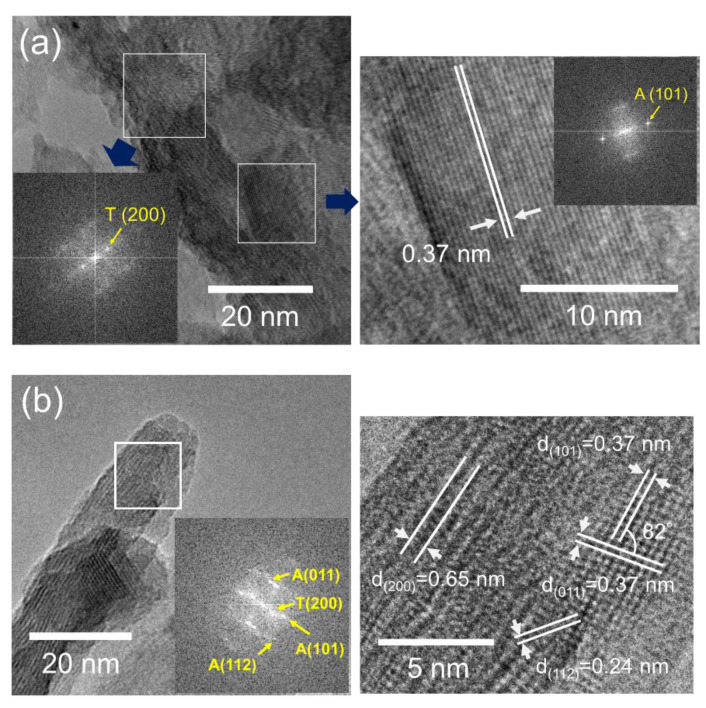
TEM images and fast Fourier transform (FFT) diffraction patterns (T = Titanate, A = Anatase) of annealed PTNT at (**a**) 400 and (**b**) 500 °C for 1 h, respectively.

Figure 6 shows the HR-TEM images and the FFT diffraction pattern of the annealed PTNTs at 400 and 500 °C. The *d*-values of the lattice fringes originated from diffraction spots which were calculated from the FFT analysis of the TEM images; they were compared with the PDF card information for hydrogen titanate (# 00-047-0124) and anatase (# 00-021-1272). In the case of the PTNTs sample annealed at 400 °C, as shown in Figure 6a, the calculated diffraction spots for one region was estimated to be the 200 plane of the hydrogen titanate with *d*-value of 0.7 nm. On the other hand, a lattice fringe with a *d*-value of 0.37 nm was also observed in the same sample (enlarged image and an FFT pattern in Figure 6a), which corresponds to the 101 plane of the anatase, in accordance with XRD results, as shown in Figure 4. As was evident from the XRD analysis results, the annealed PTNTs at 400 °C possess low crystallinity; the lattice fringes and diffraction spots assigned to only the main 101 plane of anatase were expected to be observable, while other spots (e.g., corresponding to 011, 112) are not expected to be observed.

At an annealing temperature of 500 °C, as shown in Figure 6b, two kinds of crystalline phases were also identified. The FFT diffraction spots were estimated as (011), (101), and (112) planes of the anatase as well as (200) plane of the hydrogen titanate. The enlarged image clearly shows the lattice fringes of 0.65, 0.37, 0.37, and 0.24 nm, which are assigned to 200 of hydrogen titanate and 101, 011, and 112 of anatase, respectively. These results are consistent with the results of the in situ XRD as mentioned earlier in Figure 4. As earlier discussed, the sample annealed at 500 °C exhibited a rod-like morphology with a width of 20–50 nm (see square dotted frames in Figure 2e). This rod-like PTNTs contained the two crystalline structures of hydrogen titanate and anatase. This analysis reveals that the 101 plane of anatase is parallel to the 200 plane of hydrogen titanate, and thus it is believed that the crystallites of anatase were converted directly from the peroxo-modified titanate nanotubes by the dehydration and morphological development as mentioned earlier and shown in Figure 2f.

### 3.5. Bonding Characteristics

**Figure 7:**
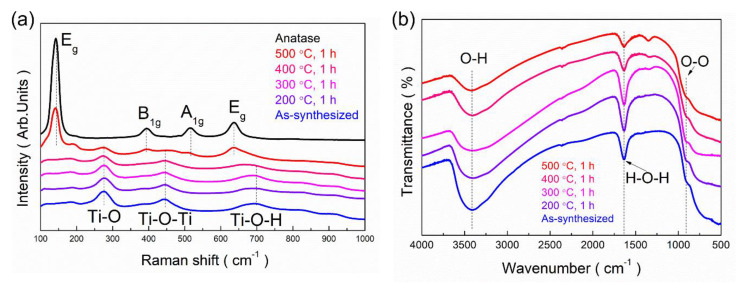
(**a**) Raman spectra and (**b**) Fourier transform infrared (FT-IR) spectra of the as-synthesized PTNTs and annealed PTNTs at different temperatures.

Raman spectroscopy and FT-IR analysis were carried out to investigate the effect of annealing temperature on the structures. Figure 7a shows the Raman spectra of structures annealed at various temperatures from 200 to 500 °C for 1 h. The Raman pattern of as-synthesized PTNTs showed a hydrogen titanate structure [46]. The peaks assigned to Ti–O, Ti–O–Ti, and Ti–O–H were observed at 276, 444, and 700 cm^−1^, respectively. After annealing at 500 °C, the intensity of peaks assigned to Ti–O and Ti–O–Ti of hydrogen titanate structure decreased, and several new peaks surfaced at 135, 395, 510, and 650 cm^−1^. These peaks were assigned to E_g_, B_1g_, A_1g_, and E_g_ of anatase. The Raman spectrum of the PTNTs annealed at 500 °C mainly consisted of the pattern of anatase, while weak peaks of hydrogen titanate structures were also confirmed.

The annealing effect on the PTNTs was further examined using a local structure-determining probe, XAFS, as shown in Figure S1. Figure S1a shows the Ti K-edge XANES spectra of the as-synthesized PTNTs and annealed PTNTs at various temperatures with anatase-type TiO_2_ as the reference sample, which demonstrate structural differences in the local structure and the electronic state of Ti^4+^ ions. Figure 7b shows the FT-IR spectra of samples obtained at the various annealing temperatures. These spectra were quite similar and were characterized by a broad and strong band at 3400 cm^−1^, which can be assigned to OH groups [47] as well as to the H_2_O molecule. The existence of this peak indicates the hydroxyl group and water molecules existed in the structure or in the interlayer space between the crystal layers. The peak intensity decreased with an increase in the annealing temperature, which led to the evaporation of water molecules and the elimination of hydroxyl groups by a dehydration reaction. This is also supported by the findings from the TG-DTA analysis shown in Figure 1. The presence of water molecules in the sample was also identified by the peak located at 1630 cm^−1^, which is assigned to the H–O–H deformation mode (δ_H–O–H_). Additionally, in all samples, a weak peak assigned to peroxo bond (–O–O–) was observed at approximately 915 cm^−1^. The intensity of the peak was slightly reduced by the heat treatment but the peak was still present even for the sample annealed at 500 °C. This sample mainly consists of anatase crystal as mentioned earlier; however, it still contains hydrogen titanate. Thus it could be inferred that the peroxo bond found in the FT-IR spectrum might still exist in the PTNTs samples annealed at 500 °C. In addition, it can also be confirmed by the X-ray photoelectron spectroscopy (XPS) analysis. The O_1s_ XPS spectrum of as-synthesized PTNTs had Ti–O–O (peroxo-) bond at 533.1 eV [24]. As can be seen in Figure S3, this peroxo-bond still existed at 533.4 eV in the PTNTs samples annealed at 500 °C with slightly decreased intensity, implying the thermal stability of the peroxo-bond in the present PTNTs.

### 3.6. Optical Properties of Materials

**Figure 8:**
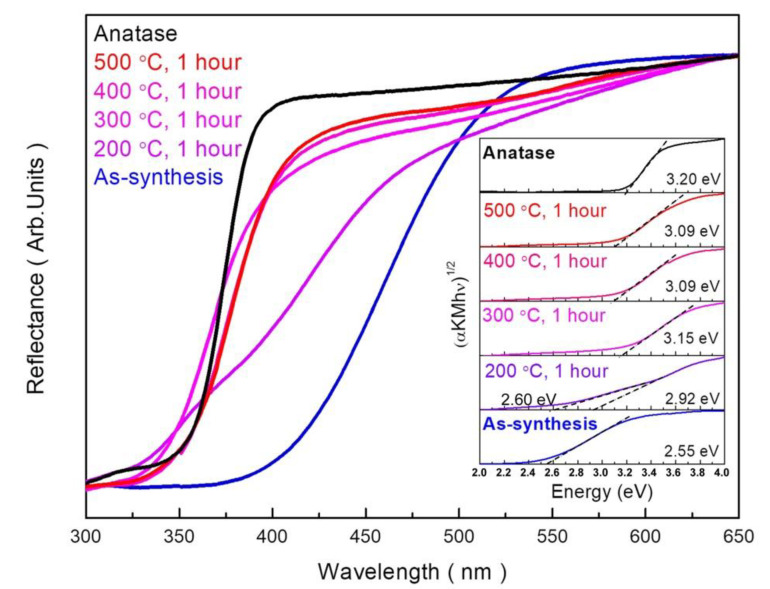
Reflectance spectra of PTNTs annealed at different temperatures. (Inset) Plots of (αhv)^1/2^ vs. energy for the annealed PTNTs at different temperatures.

The effects of annealing temperature on the optical properties and electrical band gap of the samples were studied using UV-Vis spectroscopy analysis. Figure 8 shows the reflectivity of the samples annealed at various temperatures. The UV-Vis spectrum of the as-synthesized PTNTs clearly indicates that the absorption edge located in the visible light regions is largely red-shifted compared with common anatase TiO_2_. This corresponds to the fact that the as-synthesized PTNTs are yellow. As the annealing temperature increases, the reflectivity of the incident light in the wavelength range of 350 to 500 nm increases. An increase in reflectivity is expected to reduce the absorption of light due to an increase in bandgap energy. The inset in Figure 8 show the Tauc plot for determining the optical band gap of samples calculated by the Kubelka–Munk model. The bandgap energy of the as-synthesized PTNTs was 2.55 eV, and the value increased to 3.09 eV at an annealing temperature of 500 °C. The variation of the bandgap energy was related to the presence of the peroxo bond and crystal transformation. Recently, several research groups [11,24] have shown that the bandgap energy of TiO_2_ and related compounds can be reduced due to the presence of the peroxo bond in titanate structures that increase the valance band level. However, the bandgap energy was increased by annealing, as shown in Figure 8, which might be due to the decomposition of the peroxo bond. Savinkina et al. [48] reported that the peroxo bond is stable at room temperature in the air but it can be destroyed by annealing above of 200 °C, and this phenomenon was also observed in this study as mentioned above.

However, for the 200 °C annealed sample in the present study, two kinds of optical absorption edges can be seen in the reflectance curve around 360 nm and 420 nm. These can also be found as the two linear regions in the Tauc plot, and correspond to the band gap energy of 2.92 eV and 2.60 eV, respectively. The latter value is closed to that of as-synthesized PTNT (2.55 eV) that contains –O–O– bonding, while the former one is more likely to those of annealed at higher temperature (above 300 °C). By considering these facts, we speculated that the sample annealed at 200 °C may contain two kinds of phases; one is similar to as-synthesized PTNT and the other was more likely to anatase phase. Therefore, it is considered that the crystalline phase transformation, in another words nucleation of anatase-based crystal, might have started around 200 °C, and thus two kinds of optical nature might be seen in the sample annealed at this temperature.

According to the above mentioned discussion, it has been established that annealing caused the decomposition of peroxo bond from PTNTs and it is expected to result in a decrease in the valance band level due to the increase in the titanium electron density. The XRD results showed that the hydrogen titanate crystal of PTNTs transforms into an anatase crystal structure by annealing at a temperature of 500 °C (Figure 4). At the same time, the TEM observation and Raman spectrum indicated that an anatase phase in a sample was formed at 500 °C with partial hydrogen titanate structures (Figure 6 and Figure 7). The reflectance spectrum of the sample annealed at 500 °C showed a lower reflectivity in the visible light range compared to pure anatase. Furthermore, the bandgap was calculated to be 3.09 eV which is lower than that of anatase (3.2 eV). By combining the reflectance spectrum with FT-IR analysis (Figure 7), although the peroxo bond was affected by annealing, the peroxo bond that exists in the crystal of PTNTs had high stability.

### 3.7. Formation Mechanism of Peroxo-Modified Anatase Crystal

**Figure 9:**
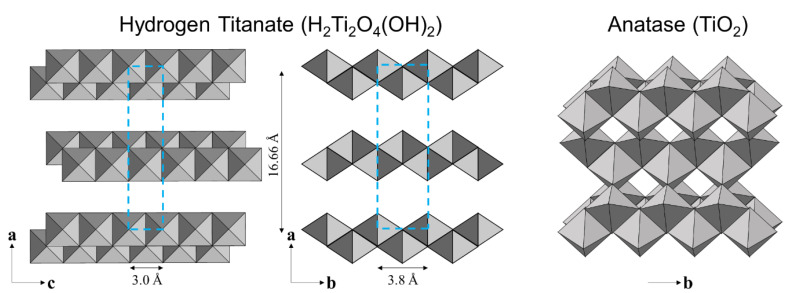
Hypothetical schematic view of a hydrogen titanate crystal.

When the crystallographic structure is considered, the H_2_Ti_2_O_5_·H_2_O (or expressed also as H_2_Ti_2_O_4_(OH)_2_) structure, which is hydrogen di-titanate, has been suggested to comprise of two-dimensional layers in which TiO_6_ octahedra are combined through edge sharing [34], as shown in Figure 9. The lattice is orthorhombic, and the TiO_6_ layers were laminated with an alternating interlayer cation (H^+^) along the [100] direction [49]. In the case of the nanotube structure of layered hydrogen titanate, Zhang et al. [50] reported that these layers scroll the [010] direction with the tube axis pointing towards the [001] direction. The configuration of the titanate layer, as shown in Figure 9, with projection along the [001] direction of the titanate, is reported to be similar to the principal unit layer of the anatase projected along the [101] direction [49]. It is hypothesized that, upon annealing, the titanate layers shrink locally, by reducing the interlayer distance and transforming into the anatase crystal [47,49]. Our results as well as those of others [47,49] have shown that local shrinkage of the titanate layers on the hydrogen titanate structure to form anatase crystal structure is possible.

**Figure 10:**
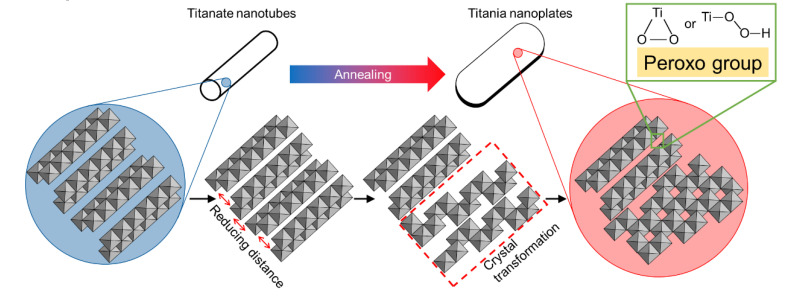
Hypothetical scheme for the transformation of titanate nanotubes to titania nanoplates. The structure models presented are the projections along the nanotube. The transformed structure shown is the anatase phase.

Figure 10 illustrates the hypothesis system for the transformation from titanate nanotubes to titania nanoplates, mimicking the lattice fringes of titanate or titania shown in the above TEM results. We observed that water, hydroxyl (–OH), and/or peroxo (–O–O–) groups were removed by dehydration reaction, which resulted from annealing that occurred in the interlayer of hydrogen titanate structure, thereby reducing the distance between that titanate layers (also see Figure 9). During this process, tubular structures with hydrogen titanate were transformed into sheet-like structures by annealing because of the removal of water from the interlayer. As discussed in the previous section and shown in Figure 2, this process may govern the morphological change by the formation of cleavage in the tubular titanate along the tube axis (crystallographic variation is illustrated in Figure 10), which results in the formation of stacked titanate nanosheets during heat treatment. Anatase crystal was then formed simultaneously along the layered hydrogen titanate by annealing, thereby titania nanoplate or rod structure that partially contained hydrogen titanate crystal was formed. Several research groups [30,31,32,34] have reported the annealing effects on TNTs, in which almost all TNTs transform into a spherical morphology with a clear anatase structure after annealing at 500 °C.

However, these previous reports are quite different from our present study. The difference in morphological change by annealing might be attributed to the peroxo groups in the interlayer of PTNTs. Our bottom-up process using the peroxo-titanium complex ion precursor was different from traditional methods [4,16,17,18] for the synthesis of nanotubular titania. During this process, the special peroxo-titanium bonds (Ti–O–O–) can be formed directly within the interlayers of the hydrogen titanate crystal as well as on the surface of materials during crystal formation without any chemical treatment [24]. Usually, the peroxo groups of TNTs are expected to be expelled by annealing. However, peroxo titanium bonds in the crystal structure of PTNTs could be restricted by the crystal transformation to anatase because this bond within the interlayer is harder to remove than that on the surface due to the difference in the heat-transfer distance from the surface to the interior [51]. Peroxo bonding was confirmed using the FT-IR of the annealed PTNTs. As a result, the peroxo group is thought to suppress the shrinkage that would have occurred between layers as a result of heat treatment. The titania nanoplates as the final products were considered to be composed of anatase crystal and layered hydrogen titanate structure with peroxo bond in their layers (Figure 10). Etacheri et al. [11] reported that oxygen vacancy accelerates the Ti–O breaking and phase transition of titania during annealing. Although a detailed analysis is required in future studies, the peroxo groups in PTNTs are expected to inhibit the generation of oxygen vacancy due to the release of oxygen by thermal decomposition. Therefore, PTNTs synthesized from Ti–O–O complex ions are also expected to partially remain in the structures due to their high thermal stability. These results indicated that the bottom-up process using ion-complex materials, which is an environmentally friendly method, is a unique method for constructing crystalline structure and morphology tunable nanostructured titanate and it has the potential to enhance the thermal stability of the nanomaterials.

## 4. Conclusions

In this study, the thermal stability of peroxo titanate nanotubes (PTNTs) synthesized by the peroxo titanium complex ion was evaluated. The as-synthesized structure was a tubular structure of hydrogen titanate H_2_Ti_2_O_5_·H_2_O that contained peroxo groups within the interlayers of the crystal structure. Dehydration and thermal decomposition by annealing were carried out in two steps: (I) Dehydration reaction of a water molecule on the surface at an annealing temperature range below 143 °C, (II) Dehydration of water and –OH groups in the crystal and transition into anatase at an annealing temperature range above 143 °C. In step I, the water loss occurred by evaporation, and in step II, dehydration reaction resulted in interstructural agglomeration and shrinkage. In addition, the transition of the interlayer of the crystal from hydrogen titanate to anatase occurred, and at the same time, the tubular structure was dismantled due to the formation of a crystal structure. The as-synthesized PTNTs had the highest surface area and it continued to decrease as the annealing temperature increased. Moreover, as the annealing temperature increased to 360 °C, anatase crystal began to form on the structure and the morphology evolved from nanotube to nanoplate-like structure along with the formation of a long axis of the nanotube structure through the transient morphology with stacked leaf-like nanosheets at an intermediate temperature that ranged from 200 °C to approximately 400 °C. The crystal structure of the nanoplate heated at 500 °C was a mixture of hydrogen titanate and anatase. Optical studies showed the extension in bandgap energies upon annealing. In addition, the results from our experiment showed that the presence of peroxo bonds within the interlayer space, which is confirmed to remain even after heat treatment at 500 °C in the mixed titanate crystal, was favors its thermal stability more than when the bond exists on the surface. Although the crystallographic properties changed with an increase in temperature above 143 °C, the relationship between the structure, morphology, optical properties of peroxo-titanate nanotubes, and the annealing temperature became clear from this study. Therefore, our findings are quite helpful for the structural design of titanate nanomaterials for various applications based on the synergy of their unique low-dimensional nanostructures and physical-chemical properties. Moreover, our visible light-activated titanate nanostructures with the high thermal stability of peroxo bond can be considered as promising material for photocatalytic and/or photovoltaic applications.

## Supplementary Materials

The following are available online at https://www.mdpi.com/2079-4991/10/7/1331/s1: Figure S1: X-ray absorption fine structure (XAFS) of anatase, as-synthesized PTNTs and annealed PTNTs at 200, 300, 400, and 500 °C for 1 h, respectively: (a) X-ray absorption near edge structure (XANES) spectra, (b) enlarged pre-edge regions, (c) enlarged white line regions, and (d) Fourier-transformed extended X-ray absorption fine structure (EXAFS) spectra. The radial distribution functions were not corrected for phase shift.
