# Supplementary material for: Effects of Annealing Temperature on the Crystal Structure, Morphology, and Optical Properties of Peroxo-Titanate Nanotubes Prepared by Peroxo-Titanium Complex Ion
Source: Nanomaterials (Basel). 2020 Jul 8;10(7):1331. doi: 10.3390/nano10071331 (PMC7407109; doi:10.3390/nano10071331)
Supplement: Supplementary file 1 [file nanomaterials-10-01331-s001.pdf]

Effects of Annealing Temperature on the Crystal Structure, Morphology, and Optical Properties of Peroxo-Titanate Nanotubes Prepared by Peroxo-Titanium Complex Ion

Hyunsu Park [1], Tomoyo Goto [1], Sunghun Cho [1], Soo Wohn Lee [2], Masato Kakihana [1,3] and Tohru Sekino [1,*]

1 Department of Advanced Hard Materials, The Institute of Scientific and Industrial Research (ISIR), Osaka University, 8-1 Mohogaoka, Osaka, Japan; hspark23@sanken.osaka-u.ac.jp (H.P.); goto@sanken.osaka-u.ac.jp (T.G.); shcho@sanken.osaka-u.ac.jp (S.H.C); kakihana@sanken.osaka-u.ac.jp (M.K.)
2 Department of Environmental and Bio-chemical Engineering, Sun Moon University, Tangjeong-Myeon, Asan, South Korea; swlee@sunmoon.ac.kr
3 Institute of Multidisciplinary Research for Advanced Materials, Tohoku University, 2-1-1 Katahira, Sendai, Japan; masato.kakihana.c1@tohoku.ac.jp
* Correspondence: sekino@sanken.osaka-u.ac.jp; Tel.: +81-(0)6-6879-8435

X-ray absorption fine structure (XAFS) analysis for synthesized PTNTs

To clarify the detailed structural change on the annealing for the synthesized PTNTs, X-ray absorption fine structure (XAFS) analysis was performed. X-ray absorption near edge structure (XANES) and extended X-ray absorption fine structure (EXAFS) spectra of Ti K-edge were obtained in an ionization chamber in transmission mode on the Kyushu University Beamline (BL06) of the Kyushu Synchrotron Light Research Centre (SAGA-LS; Tosu, Japan). Pellet samples for Ti K-edge XANES and EXAFS measurements were prepared as mixtures with boron nitride. Anatase-type TiO_2 (FUJIFILM Wako Pure Chemical Corporation, Osaka, Japan) was used as a reference sample. Ti K-edge spectra were collected over a photon energy range of 4635.2–5938.6 eV, and the spectra were analyzed using ATHENA software [1] (Ravel and Newville 2005).

Obtained XANES and EXAFS spectra for the as-synthesized and annealed PTNTs were shown in Figure S1 together with the anatase-type TiO_2 crystal.

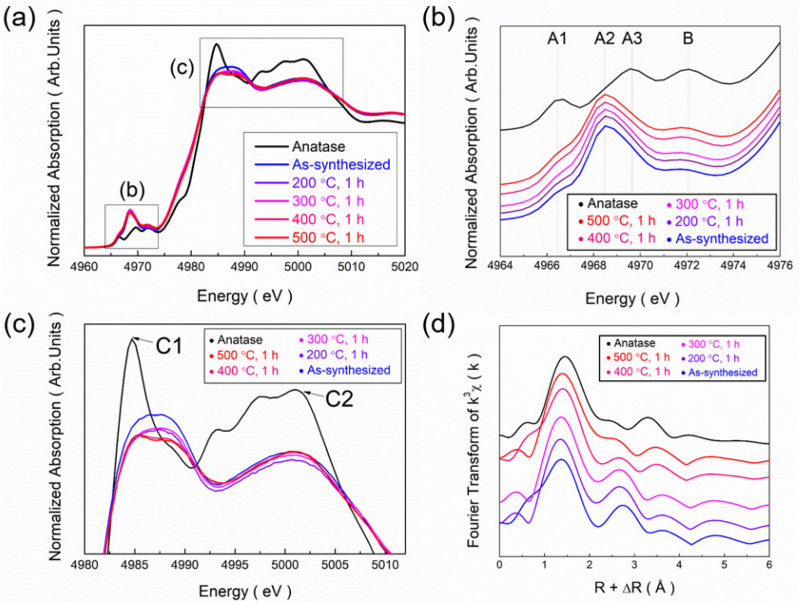

Figure S1. XAFS of anatase, as-synthesized PTNTs, and annealed PTNTs at 200, 300, 400, and 500 °C for 1 h, respectively: (a) XANES spectra, (b) enlarged pre-edge regions, (c) enlarged white line regions, and (d) Fourier transformed EXAFS spectra. The radial distribution functions were not corrected for phase shift.

Measurement of d-spacing variations by annealing temperatures

To clarify the detailed crystal structural change on the annealing for the synthesized PTNTs, the variations of d-spacing of XRD and variations in the d-spacing of TEM observation were compared and plotted in the sage graph as shown in Figure S2. As can be seen in this figure, the results were consistent and implied similar tendency.

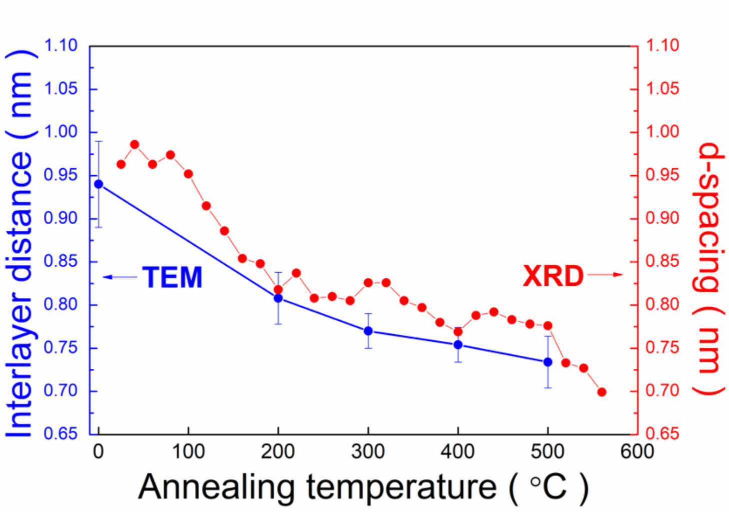

Figure S2. Variation of d-spacing measured by XRD analysis and interlayer distance obtained by the image analysis from TEM investigation for the PTNT sample by annealing. These data are re plotted from Figures 4(c) and 5(f) in the manuscript.

Investigation of peroxo-bonds

To clarify the detailed investigation of peroxo-bonds on the annealing for the synthesized PTNTs, XPS analysis for O_{1s} spectra was investigated. In the spectra of PTNTs without annealing, the peaks were separated three peaks assigned to Ti-O, H_2O, Ti-O-O bonds at 529.2, 531.6 and 533.1 eV, respectively [2]. In the case of O_{1s} spectra of annealed PTNTs at 500 °C for 1h shown in Figure S3, the same peaks assigned to Ti-O, H_2O, Ti-O-O bonds at 529.1, 531.9, and 533.4 eV, respectively. These clearly indicated the peroxo-bond was remained even after the annealing at 500 °C while the intensity of the peak (Ti-O-O bond) was slightly reduced by the annealing . This result implied high stability of peroxo-bond in the present PTNTs.

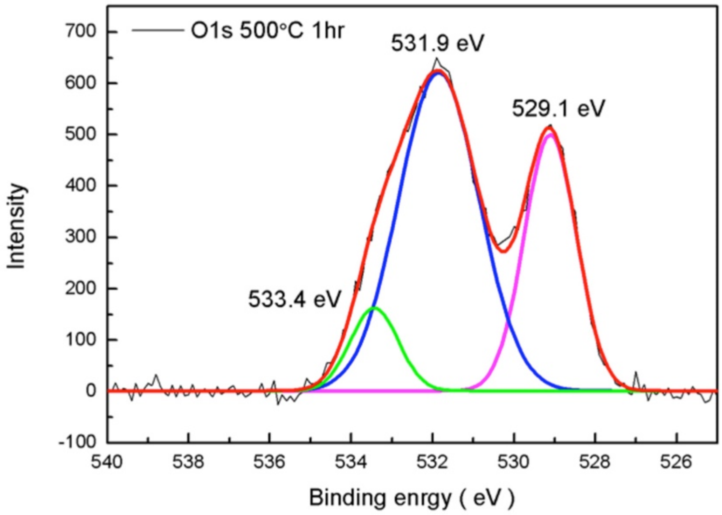

Figure S3. XPS spectrum of O_{1s} for PTNTs after annealing at 500 °C for 1h. The green, blue and pink colored lines are deconvoluted peaks from the obtained raw data.

References

1. Ravel, B.; Newville, M. ATHENA, ARTEMIS, HEPHAESTUS: Data analysis for X-ray absorption spectroscopy using IFEFFIT. *J. Synchrotron Rad.* **2005**, 12, 537–541, doi:10.1107/S0909049505012719.
2. Zou, J.; Gao, J.; Xie, F. An amorphous TiO_2 sol sensitized with H_2O_2 with the enhancement of photocatalytic activity. *J. Alloys Compd.* **2010**, *497*, 420–427, doi:10.1016/j.jallcom.2010.03.093.
